# Presbycusis: An Update on Cochlear Mechanisms and Therapies

**DOI:** 10.3390/jcm9010218

**Published:** 2020-01-14

**Authors:** Jing Wang, Jean-Luc Puel

**Affiliations:** 1 INSERM U051, Institute for Neurosciences of Montpellier, Hôpital Saint Eloi-Bâtiment INM, 80, rue Augustin Fliche-BP 74103, 34091 Montpellier, France; 2 Montpellier Neuroscience Institute, University of Montpellier, 163 rue Auguste Broussonnet, 34090 Montpellier, France

**Keywords:** age-related hearing loss, presbycusis, causal factors, mechanisms, therapies

## Abstract

Age-related hearing impairment (ARHI), also referred to as presbycusis, is the most common sensory impairment seen in the elderly. As our cochlea, the peripheral organ of hearing, ages, we tend to experience a decline in hearing and are at greater risk of cochlear sensory-neural cell degeneration and exacerbated age-related hearing impairments, e.g., gradual hearing loss, deterioration in speech comprehension (especially in noisy environments), difficulty in the localization sound sources, and ringing sensations in the ears. However, the aging process does not affect people uniformly; nor, in fact, does the aging process appear to be uniform even within an individual. Here, we outline recent research into chronological cochlear age in healthy people, and exacerbated hearing impairments during aging due to both extrinsic factors including noise and ototoxic medication, and intrinsic factors such as genetic predisposition, epigenetic factors, and aging. We review our current understanding of molecular pathways mediating ARHL and discuss recent discoveries in experimental hearing restoration and future prospects.

## 1. Introduction

Aging is a progressive decline or loss of tissue and organ function over time due to the gradual accumulation of deleterious biological changes. The aging process has three distinct components: biological degeneration, extrinsic damage, and intrinsic damage. These factors are superimposed on a genetic substrate and can be overshadowed by the general age-related susceptibility to diseases. Age-related diseases are those that are observed with increasing frequency with increasing age, such as atherosclerosis, cardiovascular disease, cancer, arthritis, cataracts, Alzheimer’s disease, presbyopia, and presbycusis. Whereas all adult humans or animals become old, not all suffer from age-related diseases. Age-related diseases can be conceptualized as accelerated aging resulting from the genetic background interacting throughout life with environmental and lifestyle factors [1].

**Figure 1:**
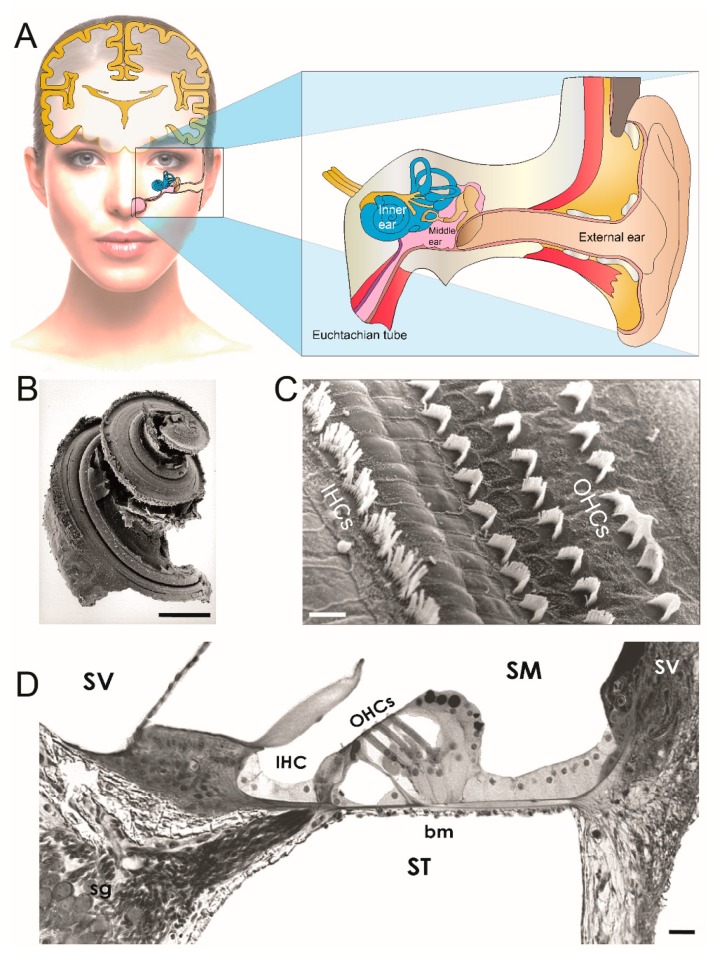
Inner-ear anatomy. (**A**) Schematic representation of ear anatomy. The ear is divided into three parts (insert): the external and middle ear transfer the sound waves to the inner ear where they are transduced into neural activity. The external ear is closed off from the middle ear by the eardrum. In the middle ear, the eardrum is mechanically linked, by a chain of three tiny bones (the ossicles), to the oval-window membrane which closes the inner ear. Embedded in the temporal bone, the inner ear comprises the balance organ or vestibule, and the hearing organ or cochlea. (**B**) Scanning electron micrograph of the organ of Corti. The cochlea is a coiled organ that forms a spiral. Scanning electron micrographs show a narrow, linear shape of IHC stereocilial bundles and a V-shape of OHC stereocilia. (**C**) Transverse section of the basal cochlear turn under light microscopy. The cochlea is made up of three canals wrapped around a bony axis, the modiolus. These canals are the scala tympani (ST), the scala vestibuli (SV), and the scala media (SM). The ST and SV are filled with perilymph. The SM is filled with endolymph. The organ of Corti is situated on the basilar membrane (bm). (**B**) = 2 mm, (**C**) = 10 µM, (**D**) = 50 µm. IHCs: inner hair cells; OHCs: outer hair cells ((**B**–**D**) micrographs courtesy of Marc Lenoir, Inserm U1051, France).

**Figure 2:**
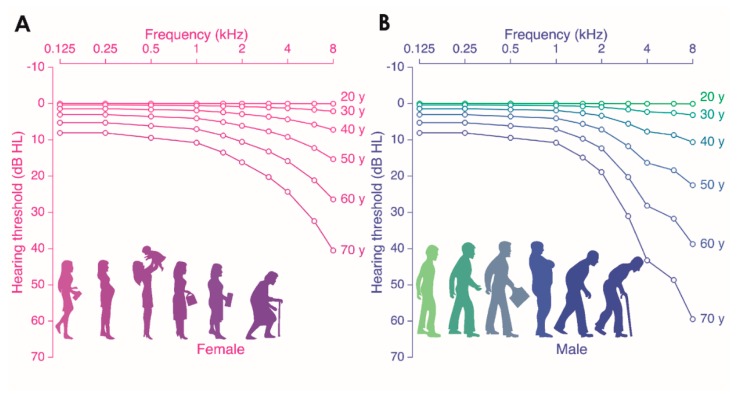
Age-related hearing loss according to the International Organization for Standardization (ISO) 7029 standard. Shown are audiograms for females (**A**) and males (**B**). The *x*-axis displays the pure tone frequency (Hz) and the *y*-axis the hearing thresholds (dB HL). Each individual graph is representative of the median audiogram at a particular age (ranging from 20 to 70 years old, with increments of 10 years).

Presbycusis, or age-related hearing loss (ARHL), is the loss of hearing that gradually occurs in most people as they grow older. According to the World Health Organization [2], approximately one third of people over 65 years of age are affected by disabling hearing loss. In 2025, there will be 1.2 billion people over 60 years of age worldwide, with more than 500 million individuals who will suffer significant impairment from presbycusis [2]. ARHL is a progressive, irreversible, and symmetrical bilateral neuro-sensory hearing loss resulting either from degeneration of the cochlea, where sound-induced vibrations are encoded by sensory hair cells into electrical signals in cochlear neurons that relay the information to the brain (Figure 1), or loss of auditory nerve fibers during cochlear aging. Hearing loss begins in the high-frequency region of the auditory spectrum and spreads towards the low-frequency regions with age (Figure 2). The age-dependent deterioration in threshold sensitivity is generally associated with difficulty in speech discrimination, as well as in sound detection and localization, particularly in noise. Males are generally more severely affected than females (Figure 2). Untreated presbycusis can contribute to social isolation, depression, and dementia [3].

Epidemiologic studies in large populations of unscreened elderly adults show that the decline in hearing sensitivity accelerates above the age of 20 to 30 in men, and above age 50 in women [4,5]. The average hearing thresholds of men exhibited a sharply rise of hearing loss in the high frequency range, whereas women’s audiograms display a more gradual sloping [4,5]. Interestingly, a high proportion of participants reported exposure to noise, otologic disease, and ototoxicity [4], suggesting that the source of the hearing impairment among unscreened populations is not exclusively associated with aging.

Based on temporal bone analyses correlating the patterns of hearing loss with defect location, Schuknecht [6] proposed three major forms of ARHL: (i) sensory presbycusis characterized by an abrupt pure-tone threshold elevation in the high frequencies and hair-cell loss at the basal end of the cochlea; (ii) strial presbycusis found in patients with a flat- or slightly descending pure-tone audiogram, correlated with atrophy of the stria vascularis; and (iii) neural presbycusis, characterized by a loss of cochlear neurons throughout the entire cochlea. The precise mechanisms underlying the age-related degeneration of the different cochlear structures remain unclear [7]. This is in part due to the complexity of each causal factor, but more importantly to the interaction of the different mechanistic pathways that can cause age-related hearing loss. Sensory hair cells are susceptible to an accumulation of injuries inflicted over time from a number of different sources, including direct mechanical, mitochondrial oxidative injury from noise, ototoxic drugs such as aminoglycosides, cisplatin, or other unknown factors [8]. The degeneration of spiral ganglion neurons (SGNs) may be triggered by the accumulation of multitudinous noise-induced loss of the afferent dendrites [9,10]. Interestingly, the most vulnerable cochlear neurons, to both noise and aging, are those with high thresholds and low spontaneous rates. Although these low-spontaneous rate (SR) fibers do not contribute to threshold detection in quiet situations, they contribute to coding of transient stimuli in the presence of continuous background noise [11], leading to the new concept called hidden hearing loss. Therefore, tone in noise detection may be a useful measure in detecting age-related hearing deficits for those patients who express difficulty, but have relatively normal thresholds in quiet [12].

This review recapitulates our current understanding of biological cochlear aging on hearing, extrinsic and intrinsic risk factors that exacerbate age-related hearing function decline in animal models and in humans, the molecular pathways mediating cochlear cell senescence and degeneration as a consequence of aging, injury (noise, ototoxic drugs), and genetic predisposition. We discuss also the recent discoveries in experimental hearing restoration.

## 2. Major Causal Factors of Age-Related Hearing Loss

The clinical presentation of presbycusis, the rate of the progression, age at onset, and ultimate severity of hearing loss varies from patient to patient. Whereas the majority of elderly patients present clear hearing losses, a significant fraction of the geriatric population has almost normal hearing. This is due to intrinsic (genetic predisposition, epigenetic factors, and aging), and extrinsic factors (e.g., noise- or ototoxic drug-exposure, head trauma, cigarette smoking) that are either the sole etiology for hearing loss, or several work in synergy with the physiopathology of presbycusis [13].

### 2.1. Biological Aging on Hearing

#### 2.1.1. Aging and Hearing in Healthy People

The clinical diagnosis of presbycusis is based on bilateral progressive loss of hearing starting from a high-frequency region of the hearing spectrum. Loss of hearing can begin in young adulthood, but is initially evident at 60 years for most people (Figure 2). Over time, the threshold elevation progresses to lower and lower frequency areas. However, presbycusis studies in humans are limited by the genetic heterogeneity and the difficulty in controlling deleterious auditory exposures over time. Despite these limitations, it has been reported that in a cohort unscreened for noise exposure, ototoxic drug exposure, and otologic disease history, presbycusis develops earlier and to a greater extent than in a highly screened cohort (without history of significant noise exposure or diseases that affect the ear) [14]. It has been suggested that the onset of hearing loss induced by biological aging is very late. Indeed, the Mabaan tribe living in the Sudanese desert retains their hearing into old age [15]. Because the hearing of the young Mabaans was the same as those of young people from other countries, the good preservation of hearing in the tribe has been attributed to their quiet living environment and generally healthy condition [16]. However, it can be argued that this difference might be caused by genetic differences between the populations. To answer this question, Goycoolea et al. [17] compared the hearing of natives of Easter Island, people living in a pre-industrial society, with those who had emigrated to Chile and spent varying amounts of time in modern society. Results showed that hearing in males that had lived or were living in Chile was significantly worse than that of males who had lived their entire lives on Easter Island, and that the poorer hearing was related to the number of years lived in modern society. Contrary to these early investigations, more recent studies showed that hearing thresholds decline with age and the rate of decline accelerates with age in presbycusis patients without noise-exposure or diseases that may affect the ear [18]. In addition, the differences of hearing thresholds between presbycusis patients with or without noise exposure are limited [19]. These results thus supported the belief that age is one of the major causal factors of ARHL.

#### 2.1.2. Aging and Hearing in Animals

To study the impact of cochlear aging on hearing, animal models are a useful tool due to their short lifespan, controlled environments and diet composition, and limited genetic heterogeneity. Gerbils that grew up in quiet environments [20] showed various degrees of threshold shifts with age. The threshold shift profile was a relatively flat loss across low and mid frequencies, with the greatest losses at the higher frequencies resembling that often seen in human presbycusis [21]. These animals also showed a decline of the endocochlear potential [22,23] and reduced amplitudes of compound action potentials of the auditory nerve [24]. Reduced amplitudes of compound action potentials in aging ears suggested asynchronous or poorly synchronized neural activity in the auditory nerve of quiet-aged gerbils [24]. Cochlear morphological examination of gerbils raised in quiet demonstrated that the most important age-related degeneration site is the stria vascularis [7]. The degeneration of marginal and intermediate cells of the stria vascularis began in both the base and apex of the cochlea, extending to the mid-cochlear regions as age increased. In addition, there was a loss of Na-K-ATPase [25] and losses of the strial capillary area in aged animals [26]. Certainly, more work with other species aged in quiet is needed in this area. However, existing data from quiet-aged gerbils make it clear that in gerbils, cochlear aging impacts specifically the stria vascularis and probably the neural structures.

### 2.2. Genetic Predispositions

Presbycusis shows a clear familial association. Heritability studies of presbycusis in humans have estimated that 25% to 75% of the variance in this pathology has a genetic component [13,27,28]. Genetic polymorphisms in the genes coding detoxification enzymes, such as glutathion S-transferase (*GSTM1* and the *GSTT1* null genotypes) and N-acetyltransferase 2 (NAT2*6A) [29,30,31] were reported to be linked to ARHL. SOD2 promoter variants (−38C > G) of the SOD2 gene encoding a ubiquitous mitochondrial superoxide dismutase enzyme (MnSOD) may link to the ARHL risk in men [32]. The main function of uncoupling protein 2 (UCP2) is the control of mitochondria-derived oxygen species (ROS) [33]. In a Japanese population, *UCP2* Ala55Val polymorphisms exhibited a significant association with ARHL [34].

An increased individual susceptibility to ARHL may rely on single nucleotide polymorphisms in the grainyhead-like 2 gene (*GRHL2*), nonsyndromic sensorineural deafness type *5* (*DFNA5*) and potassium voltage-gated channel subfamily q Member 4 (*KCNQ4*) genes, whose mutations are responsible for DFNA28, DFNA2, and DFNA5, respectively [35,36,37], but also in the glutamate metabotrophic receptor 7 gene (*GRM7*, e.g., OMIM ID: 604101) [38,39]. Finally, a common mtDNA 4977-bp deletion was frequently found in presbycusic patients [35].

Some genes associated with ARHL have also been identified in mice, including *age-related hearing* loss gene 1 (*Ahl1*), localized in chromosome 10, *Ahl2* [40] on chromosome 5 (associated with early-onset hearing loss when combined with a homozygous disease allele at the *Ahl1* locus), and *Ahl3* on chromosome 17 [41]. The Ahl candidate region contains several interesting candidate genes, including genes encoding gap-junction proteins and several collagens. Mouse strains exhibiting ARHL are also more sensitive to noise-induced hearing loss than are other strains. Collectively, polymorphisms in some monogenic deafness-causing genes, neurotransmitter-related genes, and genes involved in detoxification of oxidative stress and mitochondrial function are clearly associated with ARHL.

### 2.3. Epigenetic Factors

Traditionally, genetics and adult lifestyle factors are considered to be among the main determinants of aging-associated pathological conditions. Accumulating evidence, however, suggests that epigenetic factors may contribute to these conditions [42,43]. The term epigenetics is defined as a change in phenotype that is not caused by a change in DNA sequence [44]. Epigenetic regulation of gene expression may change over time due to environmental exposures in common complex traits. The two most well understood mechanisms of epigenetic alterations that lead to these phenotypic changes are DNA methylation and histone modifications.

#### 2.3.1. DNA Methylation

Age-related changes in DNA methylation include global hypomethylation and region-specific hypermethylation [45]. In the cochlea, the first evidence showing that involvement of aberrant DNA methylation in presbycusis came from a study focused on the gap junction protein b-2 (GJB2), in the cochlea of mimetic aging rats. In this study, Wu et al. [46] showed that hypermethylation of the promoter region of GJB2 gene resulted in connexin 26 down-regulation and an increased risk for presbycusis. Furthermore, Xu et al. [47] reported that hypermethylation of hearing-loss genes such as solute carrier family 26 member 4 (*SLC26A4*, DFNB4) and purinergic receptor P2X 2 (*P2RX2*, DFNA41) resulted in an increased risk for presbycusis in men. In addition, reduced expression of P2RX2, KCNQ5, ERBB3, and SOCS3 genes through DNA hypermethylation in elderly women was associated with presbycusis [48]. More recently, Bouzid et al. demonstrated that hypermethylation of CpG site in the cadherin-23 (*CDH23*) gene is likely to be associated with presbycusis in elderly women [48]. These results implicate complex pathogenic mechanisms underlying ARHL.

#### 2.3.2. Histone Modification

Histone proteins including H1/H5, H2A, H2B, H3, and H4 are the chief proteins of chromatin and play an important role in maintaining the shape and structure of a nucleosome. In the last few years, the role of histone modifications in aging and age-related diseases has emerged. Watanabe and Bloch [49] investigated the modification of histones in the aged cochlea of mice using immunohistochemistry. Acetylated histone H3 was detected in the spiral ganglion cells and the organ of Corti of young cochlea, but not in those of aged cochlea. Conversely, dimethylatedhistone H3 was detected in the aged group, but not in the young group. The degeneration was severest in the spira lganglion cells and the organ of Corti of the basal turn. These results suggested that histone modifications may be involved in cochlear aging regulation.

### 2.4. Environmental Factors

The complexity of etiological factors for presbycusis begins with the number of environmental risk factors, such as occupational or leisure noise, ototoxic medication (aminoglycoside, cisplatin, salicylate, loop diuretics…), cigarette smoking, and alcohol abuse [50]. However, to date, it is not clear whether these environmental factors produce some kind of early onset and/or accelerated progression of cochlear aging or whether they act on specific pathophysiological mechanisms. In this part of our review, we will focus on the two most-studied environmental factors: noise exposure and ototoxic medication.

#### 2.4.1. Noise Exposure

A retrospective clinical study from a large cohort of men in the Framingham Heart Study observed that in ears with presumed cochlear damage from previous noise exposure, subsequent progression of ARHL was exacerbated at frequencies outside the original noise-induced hearing loss [51]. These observations suggest an age-noise interaction that exacerbates age-related hearing loss in previously noise-damaged ears.

Increasing evidence from animal aging models indicates that early noise exposure renders the inner ears significantly more vulnerable to aging and may have an impact on the onset and/or progression of ARHL [9,52,53]. Indeed, Kujawa and Liberman [52] found that noise exposure in young CBA/CaJ mice, an inbred mouse strain used as “good hearing” mouse model, could trigger progressive neuronal loss and exacerbate the ARHL. Furthermore, Fernandez et al. [9] showed that interactions between noise and aging might require an acute synaptopathy to accelerate cochlear aging. In addition, repeated exposure to a short duration sound (1 h/110 dB SPL) over a long period also led to an early onset of ARHL (at six months of age) in Wistar rats when compared to non-exposed rats in which the onset of ARHL was around 12 months of age [54,55]. Although the long-term effects of early noise exposure on the aging ear are poorly understood, these clinical and experimental results indicate that noise exposure may modify the onset and/or progression of ARHL, particularly for neural presbycusis.

#### 2.4.2. Ototoxic Medications

To date, the influence of other environmental risk factors such as ototoxic medications, cigarette smoking, or alcohol abuse on ARHL is less clear and often controversial. Recently, a large longitudinal cohort study (n = 3753) aimed at elucidating the association of ototoxic medications exposure with the risk of developing hearing loss during the 10-year follow-up period demonstrated that ototoxicity-age interactions may also exacerbate age-related hearing loss in older adults [56].

## 3. Molecular Mechanisms of Presbycusis

**Figure 3:**
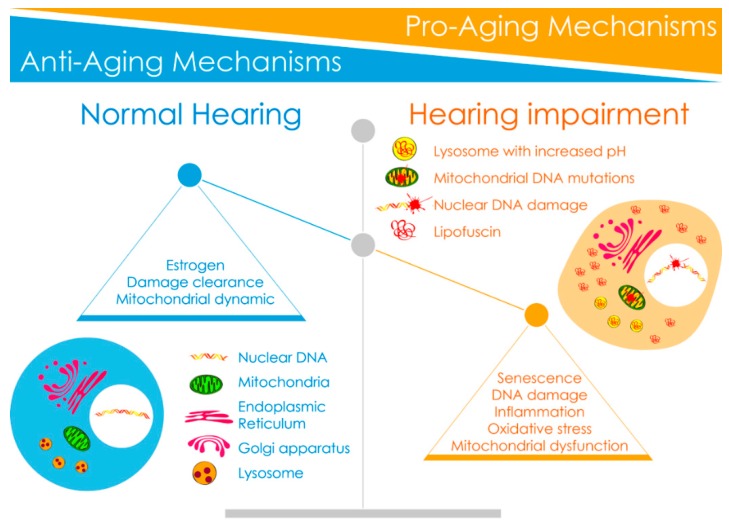
Imbalance between anti-aging and pro-aging mechanisms with age. The scheme drawing numerates several anti-aging and pro-aging mechanisms identified in the cochlear aging process. Anti-aging mechanisms include estrogen, autophagic damage clearance, and mitochondrial dynamic. Pro-aging mechanisms include oxidative stress, DNA damage, mitochondrial dysfunction, senescence-like phenotype, and senescence-associated inflammation. During the aging process, decreased activity of anti-aging molecules and increased activity of pro-aging properties might lead to accumulation of mutations in mitochondrial DNA, increased lysosomal pH with a resulting accumulation of lipofuscin and aggregates, and nuclear DNA damage, leading to cochlear cell degeneration and age-related hearing loss.

Our understanding of the molecular mechanisms associated with age-related cochlear cell degeneration and hearing loss has advanced in recent years. There are a number of pathophysiological processes that were reported to be associated with age-related changes to functional components in the inner ear. Here, we highlight recent advances in identifying various mechanisms involved in the “pro-, or anti-aging process” of the cochlea (Figure 3 and Table 1).

### 3.1. Pro-Aging Mechanisms Identified in the Cochlea

#### 3.1.1. Oxidative Stress

The oxidative stress theory of aging is based on the hypothesis that age-associated functional losses are due to the accumulation of reactive oxygen (ROS)- and nitrogen species (RNS)-induced DNA, lipid, and/or protein damage. ROS and RNS are generated by several biochemical and physiological processes of cellular metabolism. ROS and RNS are mainly generated by the mitochondria, endoplasmic reticulum, and peroxisomes under both physiological and pathological conditions. Fortunately, cells are able to counteract excessive ROS/RNS production via the activity of antioxidant enzymes such as superoxide dismutases (SODs), copper/zinc superoxide dismutase (Cu/Zn SOD), catalase, GPX (glutathione peroxidase), and GSR (glutathione reductase), as well as a variety of small-molecule antioxidants such as glutathione (GSH) and thioredoxin [57].

Increasing evidence links oxidative stress to ARHL [58,59]. Low serum levels of the ROS scavenger melatonin are significantly associated with the occurrence of high-frequency hearing loss in the elderly [60]. Experimental evidence also indicates that lipid peroxidation, oxidative DNA damage and glutathione-conjugated proteins, reduced expression of antioxidant enzymes such as catalase, and MnSOD and cytosolic SOD were associated with cochlear aging in mice [59,61,62]. In addition, mice lacking the SOD1 displayed increased age-related cochlear hair-cell loss, reduced thickness of the stria vascularis, and severe degeneration of spiral ganglion neurons [63]. By contrast, an increase in the SOD2 expression gradient in ganglion cells has been reported along the basal to apical axis of rodent and primate cochleae [64], consistent with the differential and decreased vulnerability from high-to-low frequency regions in most ARHL. Taken together, this experimental and clinical evidence indicates the potential involvement of oxidative stress in ARHL.

#### 3.1.2. DNA Damage and DNA Damage Responses

Oxidative stress may cause irreversible DNA damage [65], including adducts, single-strand breaks (SSBs), and double-strand breaks (DSBs) by base excision [66]. An early increase of 7,8-dihydro-8-oxoguanine (8-oxoG), a key biomarker of free radical-induced oxidative lesions of mitochondrial and nuclear DNA damage [67], was observed in the cytoplasm of sensory hair cells, supporting cells, and spiral ganglion neurons in the cochleae of SAMP8 mice (a cochlear premature aging model), suggesting mitochondrial DNA damage [59]. More recently, we reported that the DNA damage response could be activated in post-mitotic inner-ear cells under the stress induced by cisplatin, an anticancer drug with an ototoxic side effect [68]. A short exposure to hydrogen peroxide (H_2_O_2_) was able to induce a premature senescence phenotype in House Ear Institute-Organ of Corti 1 (HEI-OC1) mouse auditory cells. These displayed mitochondrial morphology damage, reduced mitochondrial membrane potentials, an imbalance of mitochondrial fusion/fission, and an impaired mitochondrial respiratory capacity [69]. Furthermore, using an H_2_O_2_ exposed cochlear explant in culture and adult SAMP8 mice in vivo, we demonstrated that ROS-induced DNA damage responses drive cochlear cell senescence and contribute to accelerated ARHL [70].

#### 3.1.3. Mitochondrial DNA Mutations and Dysfunction

The incidence and frequency of mtDNA point mutations and deletions increase exponentially with age and contribute to cellular senescence in humans, monkeys, and rodents, thus providing a causative link between mtDNA mutations and aging phenotypes in mammals [71]. In the cochlea, it has been hypothesized that the cochlear mitochondrial redox imbalance and mitochondrial DNA mutation and deletion might be collaboratively involved in ARHL [72]. One specific mitochondrial common deletion (mtDNA4977) is frequently observed in the temporal bone of patients with ARHL, and its measured levels are strongly correlated with the severity of ARHL [73]. Deletions and mutations in mtDNA are increased in cochleae from patients with ARHL compared to those of normal-hearing subjects [74,75]. In addition, a reduced activity of mitochondrial complex IV was observed postmortem in spiral ganglion neurons from the temporal bone of elderly patients with ARHL [76].

Mutations in *POLG*-encoding DNA polymerase γ that maintains mtDNA replication fidelity [77] or *OPA1* encoding the mitochondrial dynamin-like GTPase impact mtDNA genomic stability. These mutations are also known to cause premature hearing loss in patients and mice [78]. Mice expressing error-prone mitochondrial DNA polymerase γ (PolgD257A) defective for proof-reading activities, increasing the mutation frequency, led to the expression of defective respiratory chain proteins and premature aging [79]. These Polg knockin mice also displayed early-onset ARHL, with severe loss of SGNs and degeneration of the stria vascularis [80]. Finally, a reduced activity of complex IV was observed in cochlear tissue of SAMP8 mice aged nine months and over [59]. Thus, accumulation of mtDNA mutations and deletions may promote mitochondrial dysfunction and mitochondrial redox imbalance leading to cochlear cell aging.

#### 3.1.4. Impaired Mitochondrial Biogenesis

Mitochondrial biogenesis is the process by which cells increase their individual mitochondrial mass and copy number to increase the production of ATP as a response to greater energy expenditure, or during times of cellular stress [81]. We showed an overactive mitochondrial biogenesis in the cochlea of the senescence-accelerated mouse prone 8 strain (SAMP8) at a young age that decreased in old age; the opposite was observed in the senescence-accelerated mouse resistant 1 strain [59]. The peroxisome proliferator-activated receptor gamma coactivator 1-alpha (PGC-1α) is a key regulator of mitochondrial biogenesis. The overexpression of PGC-1α with its consequential increase in the transcription factors of nuclear respiratory factor 1 and mitochondrial transcription factor A significantly decreased the accumulation of damaged mtDNA and the number of apoptotic cells in the strial marginal cells senescence model [82].

#### 3.1.5. Senescence-Like Phenotype

In mitotic cells, cellular senescence is a permanent G1 arrest that is elicited in response to different stresses [83]. The persistence and accumulation of senescent cells has been shown to potentially play a role in the pathophysiology of aging and age-related disease [84]. Recent studies have suggested that post-mitotic cells are also capable of entering a state of senescence and exhibiting several senescence-associated properties, including heterochromatinization, synthesis of pro-inflammatory interleukins, and high senescence-associated b-galactosidase activity in the brain and retina [85,86]. Consistent with these findings, our recent results revealed that sub-lethal concentrations of hydrogen peroxide (H_2_O_2_)-exposure initiated a DNA damage response together with increased levels of key hallmarks of senescent cells, including increased expression levels of p21, and positive senescence-associated β-galactosidase labeling cells in cochlear explants in culture, together with increased inflammatory markers such as p38 and p-p38. Furthermore, high amounts of DNA damage and senescence-like features were observed in the cochlear tissues of SAMP8 mice during aging and were correlated with the accelerated ARHL observed in SAMP8 mice [70]. In addition, an accumulation of age pigment or lipofuscin was observed in the cytoplasm of spiral ganglion neurons of SAMP8 mice [59]. Together, these results suggest that post-mitotic cell senescence may be a broader phenomenon and the senescence-associated inflammatory secretory phenotype may negatively impact the functionality of cochlear cells during the aging process.

#### 3.1.6. Pro-Inflammatory Cytokines

It is now recognized that low-level chronic inflammation is correlated with the major degenerative diseases of the elderly [87]. Even the causes of the pro-inflammatory phenotype associated with aging and age-related disease are unclear; several molecular pathways have been identified that are associated with both aging and low-level inflammation. The age-related oxidative stress, the age-related accumulation of senescent cells, the senescence-associated secretory phenotype, and the decline of autophagic activity that can trigger the inflammasome [80]. In the cochlea, high levels of Interleukin-1 Beta (IL-1β) and tumor necrosis factor (TNF) have been observed in aging cochleae [59]. Furthermore, an association between systemic inflammation and ARHL has recently been noted in human studies [60,88,89]. The polymorphisms of genes encoding inflammatory mediators (TNF-α rs1800630 and TNFRSF1B rs1061624) have also been reported to contribute to the incremental risk of hearing impairment in elderly Japanese [35].

#### 3.1.7. Apoptosis

Apoptosis may play critical roles in cochlear aging and ARHL. Several apoptosis-related genes change their expression with age and with hearing loss in the CBA mouse cochlear-aging model [90]. Apoptosis and mitochondrial genome mutations are implicated in ARHL and sensory hair cell loss [91]. Deletion of the mitochondrial pro-apoptotic gene *Bak* prevented age-related loss of spiral ganglion neurons and hair cells, but also ARHL in c57BL/6J mice [62]. Consistent with these results, we also showed an increase in Bax expression and a decrease in cytochrome c oxidase expression and activity in the cochleae of SAMP8 mice during aging [51]. Finally, the ratio of the pro-apoptotic gene *BAK1*/anti-apoptotic gene *BCL2* was significantly upregulated in peripheral blood samples under ARHL and was also positively correlated with the results of the audiometric tests [92].

### 3.2. Anti-Aging Mechanisms

#### 3.2.1. Mitochondrial Quality Control and Autophagy

Mitochondrial quality control and turnover is of particular importance to cochlear sensory and neural cells because of their constant need for high levels of energy supply. Mitochondrial functionality and integrity is achieved by the quality control mechanisms of mitochondrial dynamics (fission and fusion events) and mitochondrial autophagy (mitophagy) [93].

Autophagy is a self-degradative process that plays a housekeeping role in removing misfolded or aggregated proteins and damaged organelles, as well as intracellular pathogens. The autophatic proteins Atg5, Atg4b, Atg9a, Beclin-1, and LC3B are expressed in the mouse and chicken cochlea from developmental stages through to adulthood [94]. In the adult mouse cochlea, LC3II expression was found to be primarily associated with SGNs [95], and its up-regulation occurred concomitantly with an accumulation of lipofuscin, specifically in SGNs of SAMP8 mice during the aging process [59]. It has been reported that miR-34a, which has been implicated in inducing senescence, cell cycle arrest, autophagy, and cell death [96], is also linked to ARHL in c57BL/6 mice [97]. In addition, upregulation of miR-34a in the aging cochlea was accompanied by impairment of autophagic flux, with an accumulation of phagophores and impaired autophagosome-lysosome fusion [98]. Together, these results suggest that autophagy may be a housekeeping mechanism necessary for cochlear cell survival during aging.

#### 3.2.2. Estrogen

Estrogens are the primary female sex hormones and play important roles in both reproductive and non-reproductive systems [99]. It has been reported that estrogens possess potent antioxidant properties and exert neuroprotective actions. The natural or surgical menopause is associated with mitochondrial dysfunction, neuro-inflammation, synaptic decline, cognitive impairment, and increased risk of age-related disorders [100]. Even the role of estrogen in ARHL is unclear; hearing loss is more profound in elderly males than females [101]. In addition, women with Turner’s syndrome (45, X) have estrogen deficiency as well as early onset presbycusis [102]. Finally, menopausal women who are administered hormone replacement therapy have slightly better hearing than those who are not [103]. A similar sex gap in ARHL has also been observed in aged mice and rats, as the onset of ARHL appears earlier in males than in females [104,105,106]. Together, this clinical and experimental evidence suggests that estrogen may play a protective role against ARHL.

## 4. Pharmacotherapies

Our knowledge regarding the mechanisms of cell death provides deeper insight into various cochlear diseases and thus supports the development of pharmacological therapies. This section aims to emphasize the potential importance of antioxidants, mitochondrial metabolic regulators, apoptosis inhibitors, and neurotrophins to protect cochlear cells against age-related hearing loss (Table 1).

### 4.1. Antioxidants, Free Radical Scavengers, and Anti-Inflammatories

**Figure 4:**
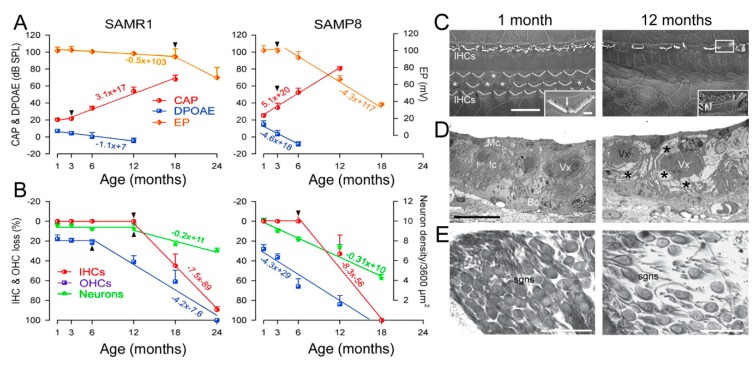
Functional and morphological assessments in SAMP8 and in SAMR1 mice. (**A**) Functional assessment. The compound action potential (CAP) threshold (red line) and distortion product otoacoustic emissions (DPOAE) amplitude (blue line) evoked by 20 kHz tone bursts, and endocochlear potential (EP) recordings (orange line, right axis)) in SAMR1 and SAMP8 mice. Fifty SAMP8 mice (n = 10 per age: 1, 3, 6, 12, 18 months) and 60 SAMR1 (n = 10 per age: 1, 3, 6, 12, 18, 24 months) mice were used for functional assessment. The lifespan of SAMR1 and SAMP8 was approximately 30 and 20 months, respectively. Note the earlier and faster increase in CAP threshold and decrease in DPOAE amplitude and EP value (arrowheads indicated broken-stick nonlinearities) in the SAMP8. In contrast to SAMR1, no CAP threshold nor DPOAEs could be recorded in 12-month-old SAMP8 mice, respectively. (**B**) Morphological assessment. Age-related loss of inner hair cells (IHCs, red line), outer hair cells (OHCs, blue line), and spiral ganglion neurons (SGNs, green line, right axis). At the end of the functional assessment period, the cochleae were removed and prepared for hair cell counting using SEM (n = 5 per age per strain) and SNG using light microscopy (n = 5 per age per strain). (**C**) Scanning electron microscopy in one and 12 months SAMP8 mice. Few OHCs are lacking (asterisks) among the three rows, but all IHCs are present at 1 month. The higher magnification insert shows an OHC stereociliary bundle with missing sterocilia (arrow). In a 12-month-old mouse, all OHCs and numerous IHCs (asterisks) have disappeared. The white box indicates a damaged IHC stereociliary bundle. In the insert, enlargement of the same IHC stereociliary bundle shows fused stereocilia. Scale bar = 10 µm; Insert in (**A**) = 1 µm. (**D**) Electron transmission microscopy of the stria vascularis. At one month, the three layers of strial cells, marginal (Mc), intermediate (Ic), basal (Bc) cells, and the blood vessels (Vx) appear normal. At 12 months, enlarged intercellular spaces and perivascular edema (asterisks) are seen. Scale bar = 10 µm. (**E**) Light microscopical evaluation of spiral ganglion loss. Shown is the normal aspect and density of neurons at 1 month, and a reduced number of spiral ganglion neurons at 12 months. Scale bar = 50 µm. (Adapted from Ménardo et al., [59]).

**Figure 5:**
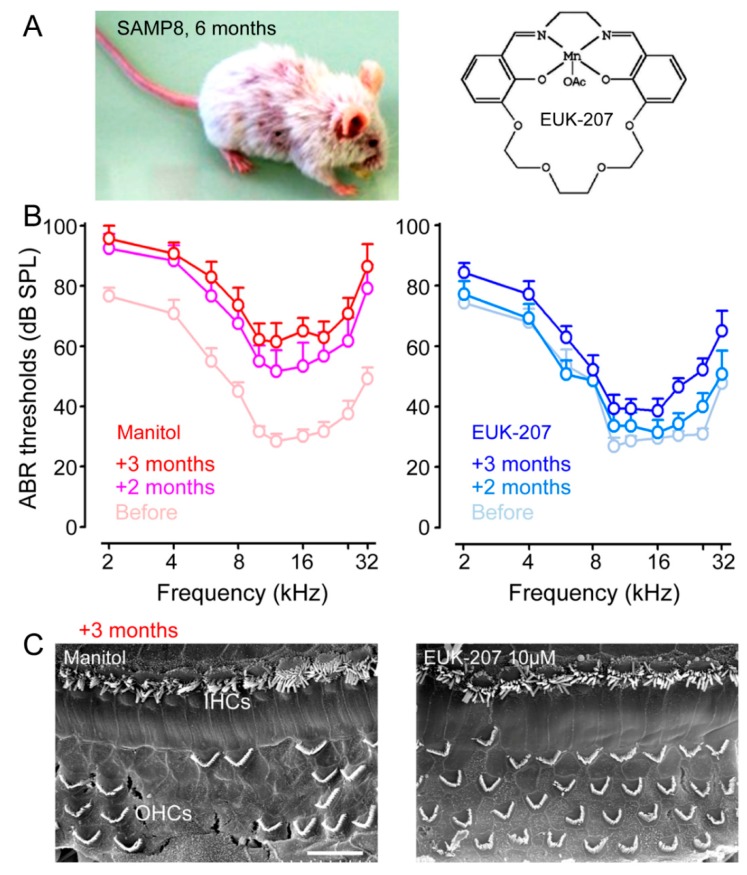
Pharmacological mitigation of ROS prevents loss of hearing and hair cells in SAMP8 mice (**A**) SAMP8 mice and EUK-207. Shown are a SAMP8 mouse aged six months and the synthetic superoxide dismutase/catalase mimetic EUK-207. (**B**) Physiological assessment. The auditory brainstem response (ABR) thresholds recorded before (pale red plot) and after two months (pink plot) and three months (red plot) of Manitol treatments, or before (pale blue plot) and after two months (azure plot) and three months (blue plot) of EUK-207 (10 µM) treatments. (**C**) Morphological assessment. Representative scanning electron micrographs showing the basal regions of cochleae from Manitol-treated (left panel) and EUK-207-treated (right panel) SAMP8 mice after three months. Scale bar = 15 µm (Adapted from Benkafadar et al. [70]).

Given the role of oxidative damage in the pathogenesis of presbycusis, antioxidants may prevent the onset or progression of ARHL. Alpha-lipoic acid is an endogenous disulfide compound synthesized de novo in mitochondria, where it is an essential enzymatic cofactor and a powerful antioxidant molecule. Alpha-lipoic acid has successfully prevented ARHL in rats [107]. Controversially, another study showed that an antioxidant-enriched diet with vitamins A, C, and E, L-carnitine, and alpha-lipoic acid significantly increased the antioxidant capacity of inner ear tissues, but did not delay the progression of presbycusis in CBA/J mice [108]. In addition, lecithin, which plays a rate-limiting role in the activation of superoxide dismutase and glutathione, also delayed the progression of presbycusis in rats by protecting cochlear mitochondrial DNA [109]. Antioxidant extracts from *Ginkgo biloba* leaves (EGb761, Renexin) prevented aging-related caspase-mediated apoptosis in rat cochleae [110]. Finally, the synthetic superoxide dismutase/catalase mimetic EUK-207 reduced age-related loss of hearing and hair-cell degeneration in senescence-accelerated mouse-prone 8 mice [70] (Figure 4 and Figure 5). Coenzyme Q10, or its analog, coenzyme Q10–Ter, which are important molecules implicated in mitochondrial energy production and protection of mitochondrial membrane proteins, lipids, and DNA from oxidative damage, prevents age-related hearing loss [62]. Even if these experimental data suggest that antioxidants might be an attractive therapy to slow ARHL, the potential protective effects of antioxidants against ARHL in elderly patients are controversial. A protective role of dietary vitamin C intake against ARHL was observed in elderly patients [111], while no protective effect against ARHL was observed in a double-blind, randomized clinical trial with EGb761 [112].

Acetylsalicylic acid (aspirin), a derivative of salicylic acid, is one of the most widely used drugs worldwide. Aspirin displays anti-inflammatory and antioxidant properties [113]. A randomized double-blind, controlled clinical trial to assess the potential therapeutic benefits of low-dose aspirin in 1262 individuals age 70 years old or older is currently underway [114].

### 4.2. Regulators of Mitochondrial Function and Metabolism

The sirtuin family has seven members (SIRT1–7) which are implicated in delaying cellular senescence and extending the organismal lifespan through the regulation of diverse cellular processes, including suppression of cellular senescence and promotion of DNA damage repair [115]. Resveratrol is a natural dietary polyphenolic SIRT1 activator that is able to mimic calorie restriction and to prolong life duration in simple organisms and experimental animal models, but is controversial in humans [116,117]. In the cochlea, dietary supplements of resveratrol significantly reduced age-related hearing loss and hair-cell loss in C57BL/6 mice [97]. Conversely, another study showed that SIRT1 deficiency reduced age-related oxidative damage of cochlear hair cells and SGNs and delayed the early onset of ARHL [118]. Caloric restriction induces upregulation of the *SIRT3* gene, which, in turn, promotes the glutathione-mediated mitochondrial antioxidant defense system and delays the onset of ARHL in mice [119]. A number of potent SIRT2 inhibitors and SIRT1 activators have now been through the first clinical trials, with evidence of safety and efficacy in psoriasis and in metabolic syndrome. This, however, is not the case for ARHL. Looking further into the future, a better understanding of the potential mechanisms of sirtuin-family effects in the cochlear aging process and ARHL could perhaps one day delay ARHL in humans with the small molecule modulators of the Sirtuins.

### 4.3. Caspase Inhibitors

A number of specific and broad-spectrum peptide caspase inhibitors have been developed to elucidate the role of each specific caspase in cell death. Intraperitoneal injection of the pan-caspase-inhibitor z-VAD-FMK Z-VAD-FMK for eight weeks, starting at one week of age in DBA/2J and A/J mice, preserved hearing by more than 10 dB SPL in the ABR thresholds and significantly reduced OHC loss in the basal turns of the cochleae [120,121]. Even many caspase inhibitors (e.g., inhibitors of caspase-1, capses-2, caspase-6, and caspase-3) have been patented for their use in the treatments of neurodegenerative diseases, cardiovascular diseases, liver diseases, and cerebral stroke. To date, however, no caspase inhibitors have entered the market due to their toxicity and poor pharmacokinetic profile [122].

### 4.4. Neurotrophins

Recent findings from temporal bones of patients aged 54 to 89 years and without an otologic disease history indicated that the degeneration of cochlear nerve peripheral axons, despite a near-normal hair-cell population, may be an important component of human presbycusis [123]. These data are consisted with previous results from the same laboratory [124] showing a decline of SGNs at a mean rate of 100 cells per year of life in human temporal bones from 100 patients aged newborn to 100 years, including only cases with normal populations of inner and outer hair cell. There were no significant gender or inter-aural differences and no significant base-to-apex gradient in degeneration. Although primary cochlear nerve degeneration is not expected to affect audiometric thresholds, it may be key to problems with hearing in noise that are characteristic of declining hearing abilities in the aging ear. Interestingly, scala tympani injection of AAV8-NT3 via cochleostomy leads to the transduction of IHCs of the basal cochlear turn, but not of the adjacent supporting cells, and prevents noise-induced synaptopathy in adult albino guinea pigs [125]. If an appropriate early diagnostic method can be identified for neural presbycusis, the regeneration of terminal nerve fibers might be a suitable therapeutic option for at least a selected group of patients.

**Table 1:** Cochlear mechanisms and therapies summarizing the pre-clinical and the clinical trials related to presbycusis with the corresponding references.

Cochlear Mechanisms of Presbycusis	Ref	Pre-Clinical and Clinical Trials (Tested Molecules)	Ref
*Oxidative stress & DNA damage* - Reactive oxygen (ROS) - Nitrogen species (RNS)	[57,58,59,60,61,62,63,64,65,66,67,68,69,70]	*Pre-clinical in animals* - Coenzyme Q10, or its analog coenzyme Q10–Ter - Superoxide dismutase/catalase mimetic EUK-207 - Alpha-lipoic acid - Cocktail vitamins A, C, and E, L-carnitine, and α-lipoic acid. - *Ginkgo biloba* (EGb761, Renexin) *Clinical trials* - Vitamin C in elderly patients, - EGb761: No protective in double-blind, randomized clinical trial.	[62,70,107,109,110,111,112]
*Mitochondrial DNA mutation & dysfunction*	[71,72,73,74,75,76,77,78,79,80,81,82]	*Pre-clinical in animals* SIRT1 activator resveratrol	[97]
*Senescence-like phenotype & Pro-inflammatory cytokines* (IL-1β and TNF)	[35,59,60,70,80,83,84,85,86,87,88,89]	*Clinical trial* Aspirin	[114]
*Apoptosis* Ratio pro-apoptotic /anti-apoptotic gene	[51,62,90,91,92]	*Pre-clinical in animals* Pan-caspase-inhibitor z-VAD-FMK Z-VAD-FMK.	[120,121]

## 5. Future Directions

Individual genetic predisposition and the physiological state of the subjects are confounding factors that are capable of influencing the success of therapeutic interventions. Of course, avoiding noise and other risk factors such as ototoxic drugs, diabetes, high blood pressure, and heart diseases can help prevent damage to cochlear hair cells which can minimize the effects of presbycusis later in life. However, this is not always possible, and treatments against presbyacusis are not still available. The complexity of the mechanisms underlying the various forms of sensory, neural, and/or striatal presbyacusis escape simple audiometric tests, underlying the need for more sophisticated tests to identify defects of cochlear functions, such as sound detection in noise. Another challenge is the need for the targeted delivery of drugs such as nanocarriers to improve the efficacy of drugs in cochlear therapies. The development of multifunction nanoparticles with a porous matrix, allowing encapsulation of a large range of molecules including chemicals, proteins, or gene products, is a promising perspective for future cochlear treatments [126]. Finally, a last but not least unresolved question concerns potential risks from the prolonged consumption of drugs. While short-term administration of antioxidants or vitamins is safe, meta-analyses of clinical trials shows that prolonged administration of beta-carotene, vitamin A, and other vitamins is associated with increased overall mortality [127]. Other therapies that may prove beneficial in the treatment of age-related hearing loss include stem-cell and gene therapies.

During the past decade, numerous efforts have been made to attempt to regrow functional hair cells or spiral ganglion neurons using adult stem cells, embryonic stem cells, and induced pluripotent stem cells in the aged inner ear. Intracerebral neural stem cell transplantation in C57BL/6J mice with presbycusis decreased apoptosis levels in the auditory cortex slightly restored auditory function and prevented further impairment of auditory function [128]. Exogenous cell transplantation in the adult and aged inner ear is more challenging due to the micro-environment of the cochlea reducing the survival rates of transplanted stem cells [93]. Several reports demonstrated that stem cells and embryonic neurons transplanted into the inner ear can survive, migrate, differentiate, and extend neuronal projections in the auditory system of adult mammals [129]. In ouabain-deafened gerbils, Corrales et al. [130] showed that transplanted otic-like neural progenitors successfully engrafted into the modiolus (central axis of the cochlea containing all the auditory nerve fibers), forming ectopic ganglia with differentiated neuronal-type cells that projected to the sensory cells in the organ of Corti. Finally, Chen et al. [131] demonstrated a restoration of auditory function after transplantation of neural progenitors in adult, ouabain-deafened animals. In the future, a new treatment for ARHI may arise from these efforts.

In a recent pioneering study, Davidsohn et al. [132] showed in mice that a single delivery of three longevity associated genes, including fibroblast growth factor 21 (FGF21), αKlotho, and a soluble form of mouse transforming growth factor-β receptor 2 (sTGFβR2) using adeno-associated viruses can mitigate four age-related diseases: obesity, type 2 diabetes, heart failure, and renal failure. These results emphasize the promise of gene therapy for the treatment of diverse age-related ailments. The interconnectedness of age-related diseases makes it worthwhile to explore the ability of these new approaches to mitigate age-related hearing loss.

In addition to pharmacological, gene, and cell therapies, progress in the technological management of presbycusis is also ongoing. Up until now, elderly individuals managed their hearing loss with hearing aids associated with listening device technologies such as frequency modulation, audio-induction loop systems, and other accessories, to coupling hearing aids with media such as phones, music players, computers, and tablet devices. However, when the hearing loss is too severe, hearing aids cannot amplify sound sufficiently, especially at the high frequencies, and this greatly compromises speech intelligibility. The remarkably improved speech discrimination observed in patients who have residual low-frequency hearing has led to the idea of extending the implantation selection criteria to patients displaying such hearing. In this context, patients with early and accelerate presbycusis may benefit from hybrid cochlear implants that combine electrical stimulation for the high-frequency loss and acoustic hearing-aid technology for the stimulation of any residual low-frequency hearing. Unfortunately, an unsolved clinical problem is the progressive loss of a patient’s residual hearing that can occur within months following cochlear implantation. Efforts to ensure the preservation of residual hearing rely on the use of soft-surgery techniques and the implantation of shorter electrode arrays to minimize the trauma caused during the process of electrode insertion [133,134]. Coating electrodes with dexamethasone significantly reduces the hearing loss caused by insertion trauma [135,136,137], limits fibrotic scars, and avoids electrical impedance increases [136,138]. In one pilot study, the safety of dexamethasone-eluting electrodes was demonstrated in a small patient group, and lower impedances were measured among the group of patients with dexamethasone-eluting cochlear implant electrodes [139].

Modern medicine is affording people longer and healthier lives. The world of artificial intelligence is quickly expanding, and hearing devices able to “learn” from our experience to address complex situations will soon be forthcoming. Moreover, the accumulation of genetic and biological biomarkers, together with classical clinical data, generates an unprecedented volume of data. These new avenues in the domain of Big Data will undoubtedly change our understanding of presbycusis, leading to molecular, individualized, patient-oriented treatments, replacing the traditional approach based on clinical symptoms and restricted laboratory or imaging markers.

## References

[1] Franceschi C., Garagnani P., Morsiani C., Conte M., Santoro A., Grignolio A., Monti D., Capri M., Salvioli S. (2018). The Continuum of Aging and Age-Related Diseases: Common Mechanisms but Different Rates. Front. Med..

[2] (2018). Addressing the Rising Prevalence of Hearing Loss.

[3] Woodcock K., Pole J.D. (2008). Educational attainment, labour force status and injury: A comparison of Canadians with and without deafness and hearing loss. Int. J. Rehabil. Res..

[4] Moscicki E.K., Elkins E.F., Baum H.M., McNamara P.M. (1985). Hearing loss in the elderly: An epidemiologic study of the Framingham Heart Study Cohort. Ear Hear..

[5] Cruickshanks K.J., Wiley T.L., Tweed T.S., Klein B.E., Klein R., Mares-Perlman J.A., Nondahl D.M. (1998). Prevalence of hearing loss in older adults in Beaver Dam, Wisconsin. The Epidemiology of Hearing Loss Study. Am. J. Epidemiol..

[6] Schuknecht H.F., Gacek M.R. (1993). Cochlear pathology in presbycusis. Ann. Otol. Rhinol. Laryngol..

[7] Gates G.A., Mills J.H. (2005). Presbycusis. Lancet.

[8] Ohlemiller K.K., Gagnon P.M. (2004). Apical-to-basal gradients in age-related cochlear degeneration and their relationship to “primary” loss of cochlear neurons. J. Comp. Neurol..

[9] Fernandez K.A., Jeffers P.W., Lall K., Liberman M.C., Kujawa S.G. (2015). Aging after noise exposure: Acceleration of cochlear synaptopathy in “recovered” ears. J. Neurosci..

[10] Kujawa S.G., Liberman M.C. (2015). Synaptopathy in the noise-exposed and aging cochlea: Primary neural degeneration in acquired sensorineural hearing loss. Hear. Res..

[11] Liberman M.C., Epstein M.J., Cleveland S.S., Wang H., Maison S.F. (2016). Toward a Differential Diagnosis of Hidden Hearing Loss in Humans. PLoS ONE.

[12] Ralli M., Greco A., De Vincentiis M., Sheppard A., Cappelli G., Neri I., Salvi R. (2019). Tone-in-noise detection deficits in elderly patients with clinically normal hearing. Am. J. Otolaryngol..

[13] Gates G.A., Couropmitree N.N., Myers R.H. (1999). Genetic associations in age-related hearing thresholds. Arch. Otolaryngol. Head Neck Surg..

[14] Guest M., Boggess M., Attia J., SHOAMP study team and Scientific Advisory Committee (2012). Relative risk of elevated hearing threshold compared to ISO1999 normative populations for Royal Australian Air Force male personnel. Hear. Res..

[15] Rosen S., Bergman M., Plester D., El-Mofty A., Satti M.H. (1962). Presbycusis study of a relatively noise-free population in the Sudan. Ann. Otol. Rhinol. Laryngol..

[16] Bergman M. (1966). Hearing in the Mabaans. A critical review of related literature. Arch. Otolaryngol..

[17] Goycoolea M.V., Goycoolea H.G., Farfan C.R., Rodriguez L.G., Martinez G.C., Vidal R. (1986). Effect of life in industrialized societies on hearing in natives of Easter Island. Laryngoscope.

[18] Bielefeld E.C., Tanaka C., Chen G.D., Henderson D. (2010). Age-related hearing loss: Is it a preventable condition?. Hear. Res..

[19] Ciorba A., Benatti A., Bianchini C., Aimoni C., Volpato S., Bovo R., Martini A. (2011). High frequency hearing loss in the elderly: Effect of age and noise exposure in an Italian group. J. Laryngol. Otol..

[20] Schmiedt R.A., Mills J.H., Adams J.C. (1990). Tuning and suppression in auditory nerve fibers of aged gerbils raised in quiet or noise. Hear. Res..

[21] Schuknecht H.F., Watanuki K., Takahashi T., Belal A.A., Kimura R.S., Jones D.D., Ota C.Y. (1974). Atrophy of the stria vascularis, a common cause for hearing loss. Laryngoscope.

[22] Gratton M.A., Schmiedt R.A., Schulte B.A. (1996). Age-related decreases in endocochlear potential are associated with vascular abnormalities in the stria vascularis. Hear. Res..

[23] Gratton M.A., Smyth B.J., Lam C.F., Boettcher F.A., Schmiedt R.A. (1997). Decline in the endocochlear potential corresponds to decreased Na,K-ATPase activity in the lateral wall of quiet-aged gerbils. Hear. Res..

[24] Hellstrom L.I., Schmiedt R.A. (1990). Compound action potential input/output functions in young and quiet-aged gerbils. Hear. Res..

[25] Schulte B.A., Schmiedt R.A. (1992). Lateral wall Na,K-ATPase and endocochlear potentials decline with age in quiet-reared gerbils. Hear. Res..

[26] Gratton M.A., Schulte B.A. (1995). Alterations in microvasculature are associated with atrophy of the stria vascularis in quiet-aged gerbils. Hear. Res..

[27] Christensen K., Frederiksen H., Hoffman H.J. (2001). Genetic and environmental influences on self-reported reduced hearing in the old and oldest old. J. Am. Geriatr. Soc..

[28] Viljanen A., Era P., Kaprio J., Pyykko I., Koskenvuo M., Rantanen T. (2007). Genetic and environmental influences on hearing in older women. J. Gerontol. Ser. A Biol. Sci. Med. Sci..

[29] Unal M., Tamer L., Dogruer Z.N., Yildirim H., Vayisoglu Y., Camdeviren H. (2005). N-acetyltransferase 2 gene polymorphism and presbycusis. Laryngoscope.

[30] Van Eyken E., Van Camp G., Fransen E., Topsakal V., Hendrickx J.J., Demeester K., Van de Heyning P., Maki-Torkko E., Hannula S., Sorri M. (2007). Contribution of the N-acetyltransferase 2 polymorphism NAT2*6A to age-related hearing impairment. J. Med. Genet..

[31] Bared A., Ouyang X., Angeli S., Du L.L., Hoang K., Yan D., Liu X.Z. (2010). Antioxidant enzymes, presbycusis, and ethnic variability. Otolaryngol. Head Neck Surg..

[32] Nolan L.S., Cadge B.A., Gomez-Dorado M., Dawson S.J. (2013). A functional and genetic analysis of SOD2 promoter variants and their contribution to age-related hearing loss. Mech. Ageing Dev..

[33] Arsenijevic D., Onuma H., Pecqueur C., Raimbault S., Manning B.S., Miroux B., Couplan E., Alves-Guerra M.C., Goubern M., Surwit R. (2000). Disruption of the uncoupling protein-2 gene in mice reveals a role in immunity and reactive oxygen species production. Nat. Genet..

[34] Sugiura S., Uchida Y., Nakashima T., Ando F., Shimokata H. (2010). The association between gene polymorphisms in uncoupling proteins and hearing impairment in Japanese elderly. Acta Oto-Laryngol..

[35] Uchida Y., Sugiura S., Sone M., Ueda H., Nakashima T. (2014). Progress and prospects in human genetic research into age-related hearing impairment. BioMed Res. Int..

[36] Van Laer L., Van Eyken E., Fransen E., Huyghe J.R., Topsakal V., Hendrickx J.J., Hannula S., Maki-Torkko E., Jensen M., Demeester K. (2008). The grainyhead like 2 gene (GRHL2), alias TFCP2L3, is associated with age-related hearing impairment. Hum. Mol. Genet..

[37] Van Eyken E., Van Laer L., Fransen E., Topsakal V., Lemkens N., Laureys W., Nelissen N., Vandevelde A., Wienker T., Van De Heyning P. (2006). KCNQ4: A gene for age-related hearing impairment?. Hum. Mutat..

[38] Friedman R.A., Van Laer L., Huentelman M.J., Sheth S.S., Van Eyken E., Corneveaux J.J., Tembe W.D., Halperin R.F., Thorburn A.Q., Thys S. (2009). GRM7 variants confer susceptibility to age-related hearing impairment. Hum. Mol. Genet..

[39] Newman D.L., Fisher L.M., Ohmen J., Parody R., Fong C.T., Frisina S.T., Mapes F., Eddins D.A., Robert Frisina D., Frisina R.D. (2012). GRM7 variants associated with age-related hearing loss based on auditory perception. Hear. Res..

[40] Johnson K.R., Zheng Q.Y. (2002). *Ahl2*, a second locus affecting age-related hearing loss in mice. Genomics.

[41] Morita Y., Hirokawa S., Kikkawa Y., Nomura T., Yonekawa H., Shiroishi T., Takahashi S., Kominami R. (2007). Fine mapping of *Ahl3* affecting both age-related and noise-induced hearing loss. Biochem. Biophys. Res. Commun..

[42] Vaiserman A., Lushchak O. (2019). Developmental origins of type 2 diabetes: Focus on epigenetics. Ageing Res. Rev..

[43] Pal S., Tyler J.K. (2016). Epigenetics and aging. Sci. Adv..

[44] Provenzano M.J., Domann F.E. (2007). A role for epigenetics in hearing: Establishment and maintenance of auditory specific gene expression patterns. Hear. Res..

[45] Xiao F.H., Kong Q.P., Perry B., He Y.H. (2016). Progress on the role of DNA methylation in aging and longevity. Brief. Funct. Genom..

[46] Wu X., Wang Y., Sun Y., Chen S., Zhang S., Shen L., Huang X., Lin X., Kong W. (2014). Reduced expression of Connexin26 and its DNA promoter hypermethylation in the inner ear of mimetic aging rats induced by d-galactose. Biochem. Biophys. Res. Commun..

[47] Xu J., Zheng J., Shen W., Ma L., Zhao M., Wang X., Tang J., Yan J., Wu Z., Zou Z. (2017). Elevated SLC26A4 gene promoter methylation is associated with the risk of presbycusis in men. Mol. Med. Rep..

[48] Bouzid A., Smeti I., Dhouib L., Roche M., Achour I., Khalfallah A., Gibriel A.A., Charfeddine I., Ayadi H., Lachuer J. (2018). Down-expression of P2RX2, KCNQ5, ERBB3 and SOCS3 through DNA hypermethylation in elderly women with presbycusis. Biomarkers.

[49] Watanabe K., Bloch W. (2013). Histone methylation and acetylation indicates epigenetic change in the aged cochlea of mice. Eur. Arch. Oto-Rhino-Laryngol..

[50] Fransen E., Lemkens N., Van Laer L., Van Camp G. (2003). Age-related hearing impairment (ARHI): Environmental risk factors and genetic prospects. Exp. Gerontol..

[51] Gates G.A., Schmid P., Kujawa S.G., Nam B., D’Agostino R. (2000). Longitudinal threshold changes in older men with audiometric notches. Hear. Res..

[52] Kujawa S.G., Liberman M.C. (2006). Acceleration of age-related hearing loss by early noise exposure: Evidence of a misspent youth. J. Neurosci..

[53] Fetoni A.R., Picciotti P.M., Paludetti G., Troiani D. (2011). Pathogenesis of presbycusis in animal models: A review. Exp. Gerontol..

[54] Alvarado J.C., Fuentes-Santamaria V., Gabaldon-Ull M.C., Juiz J.M. (2019). Age-Related Hearing Loss Is Accelerated by Repeated Short-Duration Loud Sound Stimulation. Front. Neurosci..

[55] Alvarado J.C., Fuentes-Santamaria V., Gabaldon-Ull M.C., Blanco J.L., Juiz J.M. (2014). Wistar rats: A forgotten model of age-related hearing loss. Front. Aging Neurosci..

[56] Joo Y., Cruickshanks K.J., Klein B.E.K., Klein R., Hong O., Wallhagen M. (2019). The Contribution of Ototoxic Medications to Hearing Loss among Older Adults. J. Gerontol. Ser. A Biol. Sci. Med. Sci..

[57] Pacher P., Beckman J.S., Liaudet L. (2007). Nitric oxide and peroxynitrite in health and disease. Physiol. Rev..

[58] Han C., Someya S. (2013). Maintaining good hearing: Calorie restriction, Sirt3, and glutathione. Exp. Gerontol..

[59] Menardo J., Tang Y., Ladrech S., Lenoir M., Casas F., Michel C., Bourien J., Ruel J., Rebillard G., Maurice T. (2012). Oxidative stress, inflammation, and autophagic stress as the key mechanisms of premature age-related hearing loss in SAMP8 mouse Cochlea. Antioxid. Redox Signal..

[60] Lasisi A.O., Fehintola F.A. (2011). Correlation between plasma levels of radical scavengers and hearing threshold among elderly subjects with age-related hearing loss. Acta Oto-Laryngol..

[61] Jiang H., Talaska A.E., Schacht J., Sha S.H. (2007). Oxidative imbalance in the aging inner ear. Neurobiol. Aging.

[62] Someya S., Xu J., Kondo K., Ding D., Salvi R.J., Yamasoba T., Rabinovitch P.S., Weindruch R., Leeuwenburgh C., Tanokura M. (2009). Age-related hearing loss in C57BL/6J mice is mediated by Bak-dependent mitochondrial apoptosis. Proc. Natl. Acad. Sci. USA.

[63] Keithley E.M., Canto C., Zheng Q.Y., Wang X., Fischel-Ghodsian N., Johnson K.R. (2005). Cu/Zn superoxide dismutase and age-related hearing loss. Hear. Res..

[64] Ying Y.L., Balaban C.D. (2009). Regional distribution of manganese superoxide dismutase 2 (Mn SOD2) expression in rodent and primate spiral ganglion cells. Hear. Res..

[65] Mantha A.K., Sarkar B., Tell G. (2014). A short review on the implications of base excision repair pathway for neurons: Relevance to neurodegenerative diseases. Mitochondrion.

[66] Breen A.P., Murphy J.A. (1995). Reactions of oxyl radicals with DNA. Free Radic. Biol. Med..

[67] Valavanidis A., Vlachogianni T., Fiotakis C. (2009). 8-hydroxy-2′ -deoxyguanosine (8-OHdG): A critical biomarker of oxidative stress and carcinogenesis. J. Environ. Sci. Health C Environ. Carcinog. Ecotoxicol. Rev..

[68] Benkafadar N., Menardo J., Bourien J., Nouvian R., Francois F., Decaudin D., Maiorano D., Puel J.L., Wang J. (2017). Reversible p53 inhibition prevents cisplatin ototoxicity without blocking chemotherapeutic efficacy. EMBO Mol. Med..

[69] Kamogashira T., Hayashi K., Fujimoto C., Iwasaki S., Yamasoba T. (2017). Functionally and morphologically damaged mitochondria observed in auditory cells under senescence-inducing stress. NPJ Aging Mech. Dis..

[70] Benkafadar N., Francois F., Affortit C., Casas F., Ceccato J.C., Menardo J., Venail F., Malfroy-Camine B., Puel J.L., Wang J. (2019). ROS-Induced Activation of DNA Damage Responses Drives Senescence-Like State in Postmitotic Cochlear Cells: Implication for Hearing Preservation. Mol. Neurobiol..

[71] Trifunovic A., Wredenberg A., Falkenberg M., Spelbrink J.N., Rovio A.T., Bruder C.E., Bohlooly Y.M., Gidlof S., Oldfors A., Wibom R. (2004). Premature ageing in mice expressing defective mitochondrial DNA polymerase. Nature.

[72] Chen H., Tang J. (2014). The role of mitochondria in age-related hearing loss. Biogerontology.

[73] Markaryan A., Nelson E.G., Hinojosa R. (2009). Quantification of the mitochondrial DNA common deletion in presbycusis. Laryngoscope.

[74] Bai U., Seidman M.D., Hinojosa R., Quirk W.S. (1997). Mitochondrial DNA deletions associated with aging and possibly presbycusis: A human archival temporal bone study. Am. J. Otol..

[75] Fischel-Ghodsian N., Bykhovskaya Y., Taylor K., Kahen T., Cantor R., Ehrenman K., Smith R., Keithley E. (1997). Temporal bone analysis of patients with presbycusis reveals high frequency of mitochondrial mutations. Hear. Res..

[76] Markaryan A., Nelson E.G., Hinojosa R. (2010). Major arc mitochondrial DNA deletions in cytochrome c oxidase-deficient human cochlear spiral ganglion cells. Acta Oto-Laryngol..

[77] Filosto M., Mancuso M., Nishigaki Y., Pancrudo J., Harati Y., Gooch C., Mankodi A., Bayne L., Bonilla E., Shanske S. (2003). Clinical and genetic heterogeneity in progressive external ophthalmoplegia due to mutations in polymerase gamma. Arch. Neurol..

[78] Sarzi E., Angebault C., Seveno M., Gueguen N., Chaix B., Bielicki G., Boddaert N., Mausset-Bonnefont A.L., Cazevieille C., Rigau V. (2012). The human OPA1delTTAG mutation induces premature age-related systemic neurodegeneration in mouse. Brain.

[79] Trifunovic A., Hansson A., Wredenberg A., Rovio A.T., Dufour E., Khvorostov I., Spelbrink J.N., Wibom R., Jacobs H.T., Larsson N.G. (2005). Somatic mtDNA mutations cause aging phenotypes without affecting reactive oxygen species production. Proc. Natl. Acad. Sci. USA.

[80] Kujoth G.C., Hiona A., Pugh T.D., Someya S., Panzer K., Wohlgemuth S.E., Hofer T., Seo A.Y., Sullivan R., Jobling W.A. (2005). Mitochondrial DNA mutations, oxidative stress, and apoptosis in mammalian aging. Science.

[81] Sanchis-Gomar F., Garcia-Gimenez J.L., Gomez-Cabrera M.C., Pallardo F.V. (2014). Mitochondrial biogenesis in health and disease. Molecular and therapeutic approaches. Curr. Pharm. Des..

[82] Zhao X.Y., Sun J.L., Hu Y.J., Yang Y., Zhang W.J., Hu Y., Li J., Sun Y., Zhong Y., Peng W. (2013). The effect of overexpression of PGC-1alpha on the mtDNA4834 common deletion in a rat cochlear marginal cell senescence model. Hear. Res..

[83] Herranz N., Gil J. (2018). Mechanisms and functions of cellular senescence. J. Clin. Investig..

[84] Childs B.G., Durik M., Baker D.J., van Deursen J.M. (2015). Cellular senescence in aging and age-related disease: From mechanisms to therapy. Nat. Med..

[85] van Deursen J.M. (2014). The role of senescent cells in ageing. Nature.

[86] Sapieha P., Mallette F.A. (2018). Cellular Senescence in Postmitotic Cells: Beyond Growth Arrest. Trends Cell Biol..

[87] Howcroft T.K., Campisi J., Louis G.B., Smith M.T., Wise B., Wyss-Coray T., Augustine A.D., McElhaney J.E., Kohanski R., Sierra F. (2013). The role of inflammation in age-related disease. Aging.

[88] Nash S.D., Cruickshanks K.J., Zhan W., Tsai M.Y., Klein R., Chappell R., Nieto F.J., Klein B.E., Schubert C.R., Dalton D.S. (2014). Long-term assessment of systemic inflammation and the cumulative incidence of age-related hearing impairment in the epidemiology of hearing loss study. J. Gerontol. Ser. A Biol. Sci. Med. Sci..

[89] Verschuur C.A., Dowell A., Syddall H.E., Ntani G., Simmonds S.J., Baylis D., Gale C.R., Walsh B., Cooper C., Lord J.M. (2012). Markers of inflammatory status are associated with hearing threshold in older people: Findings from the Hertfordshire Ageing Study. Age Ageing.

[90] Tadros S.F., D’Souza M., Zhu X., Frisina R.D. (2008). Apoptosis-related genes change their expression with age and hearing loss in the mouse cochlea. Apoptosis Int. J. Program. Cell Death.

[91] Fujimoto C., Yamasoba T. (2014). Oxidative stresses and mitochondrial dysfunction in age-related hearing loss. Oxid. Med. Cell. Longev..

[92] Falah M., Houshmand M., Najafi M., Balali M., Mahmoudian S., Asghari A., Emamdjomeh H., Farhadi M. (2016). The potential role for use of mitochondrial DNA copy number as predictive biomarker in presbycusis. Ther. Clin. Risk Manag..

[93] Wang J., Puel J.L. (2018). Toward Cochlear Therapies. Physiol. Rev..

[94] Aburto M.R., Sanchez-Calderon H., Hurle J.M., Varela-Nieto I., Magarinos M. (2012). Early otic development depends on autophagy for apoptotic cell clearance and neural differentiation. Cell Death Dis..

[95] de Iriarte Rodriguez R., Pulido S., Rodriguez-de la Rosa L., Magarinos M., Varela-Nieto I. (2015). Age-regulated function of autophagy in the mouse inner ear. Hear. Res..

[96] Liu N., Landreh M., Cao K., Abe M., Hendriks G.J., Kennerdell J.R., Zhu Y., Wang L.S., Bonini N.M. (2012). The microRNA miR-34 modulates ageing and neurodegeneration in Drosophila. Nature.

[97] Xiong H., Pang J., Yang H., Dai M., Liu Y., Ou Y., Huang Q., Chen S., Zhang Z., Xu Y. (2015). Activation of miR-34a/SIRT1/p53 signaling contributes to cochlear hair cell apoptosis: Implications for age-related hearing loss. Neurobiol. Aging.

[98] Pang J., Xiong H., Lin P., Lai L., Yang H., Liu Y., Huang Q., Chen S., Ye Y., Sun Y. (2017). Activation of miR-34a impairs autophagic flux and promotes cochlear cell death via repressing ATG9A: Implications for age-related hearing loss. Cell Death Dis..

[99] Cui J., Shen Y., Li R. (2013). Estrogen synthesis and signaling pathways during aging: From periphery to brain. Trends Mol. Med..

[100] Zarate S., Stevnsner T., Gredilla R. (2017). Role of Estrogen and Other Sex Hormones in Brain Aging. Neuroprotection and DNA Repair. Front. Aging Neurosci..

[101] Jonsson R., Rosenhall U., Gause-Nilsson I., Steen B. (1998). Auditory function in 70- and 75-year-olds of four age cohorts. A cross-sectional and time-lag study of presbyacusis. Scand. Audiol..

[102] Stenberg A.E., Nylen O., Windh M., Hultcrantz M. (1998). Otological problems in children with Turner’s syndrome. Hear. Res..

[103] Hultcrantz M., Simonoska R., Stenberg A.E. (2006). Estrogen and hearing: A summary of recent investigations. Acta Oto-Laryngol..

[104] Balogova Z., Popelar J., Chiumenti F., Chumak T., Burianova J.S., Rybalko N., Syka J. (2017). Age-Related Differences in Hearing Function and Cochlear Morphology between Male and Female Fischer 344 Rats. Front. Aging Neurosci..

[105] Guimaraes P., Zhu X., Cannon T., Kim S., Frisina R.D. (2004). Sex differences in distortion product otoacoustic emissions as a function of age in CBA mice. Hear. Res..

[106] Henry K.R. (2004). Males lose hearing earlier in mouse models of late-onset age-related hearing loss; females lose hearing earlier in mouse models of early-onset hearing loss. Hear. Res..

[107] Seidman M.D., Khan M.J., Bai U., Shirwany N., Quirk W.S. (2000). Biologic activity of mitochondrial metabolites on aging and age-related hearing loss. Am. J. Otol..

[108] Sha S.H., Kanicki A., Halsey K., Wearne K.A., Schacht J. (2012). Antioxidant-enriched diet does not delay the progression of age-related hearing loss. Neurobiol. Aging.

[109] Seidman M.D., Khan M.J., Tang W.X., Quirk W.S. (2002). Influence of lecithin on mitochondrial DNA and age-related hearing loss. Otolaryngol. Head Neck Surg..

[110] Nevado J., Sanz R., Sanchez-Rodriguez C., Garcia-Berrocal J.R., Martin-Sanz E., Gonzalez-Garcia J.A., Esteban-Sanchez J., Ramirez-Camacho R. (2010). Ginkgo biloba extract (EGb761) protects against aging-related caspase-mediated apoptosis in rat cochlea. Acta Oto-Laryngol..

[111] Kang J.W., Choi H.S., Kim K., Choi J.Y. (2014). Dietary vitamin intake correlates with hearing thresholds in the older population: The Korean National Health and Nutrition Examination Survey. Am. J. Clin. Nutr..

[112] Polanski J.F., Cruz O.L. (2013). Evaluation of antioxidant treatment in presbyacusis: Prospective, placebo-controlled, double-blind, randomised trial. J. Laryngol. Otol..

[113] Esposito E., Di Matteo V., Benigno A., Pierucci M., Crescimanno G., Di Giovanni G. (2007). Non-steroidal anti-inflammatory drugs in Parkinson’s disease. Exp. Neurol..

[114] Lowthian J.A., Britt C.J., Rance G., Lin F.R., Woods R.L., Wolfe R., Nelson M.R., Dillon H.A., Ward S., Reid C.M. (2016). Slowing the progression of age-related hearing loss: Rationale and study design of the ASPIRIN in HEARING, retinal vessels imaging and neurocognition in older generations (ASPREE-HEARING) trial. Contemp. Clin. Trials.

[115] Lee S.H., Lee H.Y., Yu M., Yeom E., Lee J.H., Yoon A., Lee K.S., Min K.J. (2019). Extension of Drosophila lifespan by Korean red ginseng through a mechanism dependent on dSir2 and insulin/IGF-1 signaling. Aging.

[116] Rascon B., Hubbard B.P., Sinclair D.A., Amdam G.V. (2012). The lifespan extension effects of resveratrol are conserved in the honey bee and may be driven by a mechanism related to caloric restriction. Aging.

[117] Agarwal B., Baur J.A. (2011). Resveratrol and life extension. Ann. N. Y. Acad. Sci..

[118] Han C., Linser P., Park H.J., Kim M.J., White K., Vann J.M., Ding D., Prolla T.A., Someya S. (2016). Sirt1 deficiency protects cochlear cells and delays the early onset of age-related hearing loss in C57BL/6 mice. Neurobiol. Aging.

[119] Someya S., Tanokura M., Weindruch R., Prolla T.A., Yamasoba T. (2010). Effects of caloric restriction on age-related hearing loss in rodents and rhesus monkeys. Curr. Aging Sci..

[120] Han X., Ge R., Xie G., Li P., Zhao X., Gao L., Zhang H., Wang O., Huang F., Han F. (2015). Caspase-mediated apoptosis in the cochleae contributes to the early onset of hearing loss in A/J mice. ASN Neuro.

[121] Yang L., Zhang H., Han X., Zhao X., Hu F., Li P., Xie G., Gao L., Cheng L., Song X. (2015). Attenuation of hearing loss in DBA/2J mice by anti-apoptotic treatment. Hear. Res..

[122] Lee H., Shin E.A., Lee J.H., Ahn D., Kim C.G., Kim J.H., Kim S.H. (2018). Caspase inhibitors: A review of recently patented compounds (2013–2015). Expert Opin. Ther. Pat..

[123] Viana L.M., O’Malley J.T., Burgess B.J., Jones D.D., Oliveira C.A., Santos F., Merchant S.N., Liberman L.D., Liberman M.C. (2015). Cochlear neuropathy in human presbycusis: Confocal analysis of hidden hearing loss in post-mortem tissue. Hear. Res..

[124] Makary C.A., Shin J., Kujawa S.G., Liberman M.C., Merchant S.N. (2011). Age-related primary cochlear neuronal degeneration in human temporal bones. J. Assoc. Res. Otolaryngol..

[125] Chen H., Xing Y., Xia L., Chen Z., Yin S., Wang J. (2018). AAV-mediated NT-3 overexpression protects cochleae against noise-induced synaptopathy. Gene Ther..

[126] Pyykko I., Zou J., Schrott-Fischer A., Glueckert R., Kinnunen P. (2016). An Overview of Nanoparticle Based Delivery for Treatment of Inner Ear Disorders. Methods Mol. Biol..

[127] Miller R.A. (2005). Evaluating evidence for aging. Science.

[128] Ren H., Chen J., Wang Y., Zhang S., Zhang B. (2013). Intracerebral neural stem cell transplantation improved the auditory of mice with presbycusis. Int. J. Clin. Exp. Pathol..

[129] Hu Z., Ulfendahl M. (2006). Cell replacement therapy in the inner ear. Stem Cells Dev..

[130] Corrales C.E., Pan L., Li H., Liberman M.C., Heller S., Edge A.S. (2006). Engraftment and differentiation of embryonic stem cell-derived neural progenitor cells in the cochlear nerve trunk: Growth of processes into the organ of Corti. J. Neurobiol..

[131] Chen W., Jongkamonwiwat N., Abbas L., Eshtan S.J., Johnson S.L., Kuhn S., Milo M., Thurlow J.K., Andrews P.W., Marcotti W. (2012). Restoration of auditory evoked responses by human ES-cell-derived otic progenitors. Nature.

[132] Davidsohn N., Pezzone M., Vernet A., Graveline A., Oliver D., Slomovic S., Punthambaker S., Sun X., Liao R., Bonventre J.V. (2019). A single combination gene therapy treats multiple age-related diseases. Proc. Natl. Acad. Sci. USA.

[133] Gantz B.J., Turner C. (2004). Combining acoustic and electrical speech processing: Iowa/Nucleus hybrid implant. Acta Oto-Laryngol..

[134] Skarzynski H., Lorens A., Piotrowska A., Podskarbi-Fayette R. Results of partial deafness cochlear implantation using various electrode designs. Audiol. Neuro-Otol..

[135] Liu Y., Jolly C., Braun S., Stark T., Scherer E., Plontke S.K., Kiefer J. (2016). In vitro and in vivo pharmacokinetic study of a dexamethasone-releasing silicone for cochlear implants. Eur. Arch. Oto-Rhino-Laryngol..

[136] Douchement D., Terranti A., Lamblin J., Salleron J., Siepmann F., Siepmann J., Vincent C. (2015). Dexamethasone eluting electrodes for cochlear implantation: Effect on residual hearing. Cochlear Implant. Int..

[137] Astolfi L., Simoni E., Giarbini N., Giordano P., Pannella M., Hatzopoulos S., Martini A. (2016). Cochlear implant and inflammation reaction: Safety study of a new steroid-eluting electrode. Hear. Res..

[138] Bas E., Bohorquez J., Goncalves S., Perez E., Dinh C.T., Garnham C., Hessler R., Eshraghi A.A., Van De Water T.R. (2016). Electrode array-eluted dexamethasone protects against electrode insertion trauma induced hearing and hair cell losses, damage to neural elements, increases in impedance and fibrosis: A dose response study. Hear. Res..

[139] Plontke S.K., Gotze G., Rahne T., Liebau A. (2016). Intracochlear drug delivery in combination with cochlear implants : Current aspects. Hno.

